# Insecticide Resistance Alleles Affect Vector Competence of *Anopheles gambiae s.s.* for *Plasmodium falciparum* Field Isolates

**DOI:** 10.1371/journal.pone.0063849

**Published:** 2013-05-21

**Authors:** Haoues Alout, Nicaise Tuikue Ndam, Marcel Maurice Sandeu, Innocent Djégbe, Fabrice Chandre, Roch Kounbobr Dabiré, Luc Salako Djogbénou, Vincent Corbel, Anna Cohuet

**Affiliations:** 1 Institut de recherche pour le développement (IRD), Maladies Infectieuses et Vecteurs, Ecologie, Génétique, Evolution et Contrôle (MIVEGEC), UM1-UM2-CNRS 5290 IRD 224, Montpellier, France; 2 Institut de Recherche en Sciences de la Santé (IRSS), 01 BP 545 Bobo-Dioulasso 01, Burkina Faso; 3 Institut des Sciences Biomédicale et Appliquées, Cotonou, Bénin; 4 IRD, UMR 216, Mère et Enfant face aux Infections Tropicales, Université Paris Descartes, Paris, France; 5 Centre de Recherche Entomologique de Cotonou, Cotonou, Bénin; 6 Institut Régional de Santé Publique/Université d’Abomey-Calavi, Cotonou, Bénin; 7 Department of Entomology, Faculty of Agriculture, Kasetsart University, Bangkok, Thailand; University of Crete, Greece

## Abstract

The widespread insecticide resistance raises concerns for vector control implementation and sustainability particularly for the control of the main vector of human malaria, *Anopheles gambiae sensu stricto*. However, the extent to which insecticide resistance mechanisms interfere with the development of the malignant malaria parasite in its vector and their impact on overall malaria transmission remains unknown. We explore the impact of insecticide resistance on the outcome of *Plasmodium falciparum* infection in its natural vector using three *An. gambiae* strains sharing a common genetic background, one susceptible to insecticides and two resistant, one homozygous for the *ace-1^R^* mutation and one for the *kdr* mutation. Experimental infections of the three strains were conducted in parallel with field isolates of *P. falciparum* from Burkina Faso (West Africa) by direct membrane feeding assays. Both insecticide resistant mutations influence the outcome of malaria infection by increasing the prevalence of infection. In contrast, the *kdr* resistant allele is associated with reduced parasite burden in infected individuals at the oocyst stage, when compared to the susceptible strain, while the *ace-1* ^R^ resistant allele showing no such association. Thus insecticide resistance, which is particularly problematic for malaria control efforts, impacts vector competence towards *P. falciparum* and probably parasite transmission through increased sporozoite prevalence in *kdr* resistant mosquitoes. These results are of great concern for the epidemiology of malaria considering the widespread pyrethroid resistance currently observed in Sub-Saharan Africa and the efforts deployed to control the disease.

## Introduction

Mosquito vectors experience a very intense selective pressure from insecticides used in malaria control programs through distribution of impregnated bed nets and use of indoor-residual spraying [1] with added pressure from the heavy use of insecticides for agricultural purposes [2], [3]. As a consequence, the selection of insecticide resistance mutations has occurred in many vector species and various resistant alleles are now widespread as a result of frequent applications of organochlorines (e.g. DDT), organophosphates (OP), carbamates (CX), and pyrethroids (PYR). Two main mechanisms are responsible for insecticide resistance: increased metabolism of detoxification, which involves enhanced degradation or sequestration of insecticide molecules and modification of the insecticide target site, which becomes less sensitive to insecticide action. The molecular basis of target site insensitivity has been characterized in many insect species (reviewed in [4]) and has demonstrated conserved resistant mutations across insect vectors. In several mosquito species and populations, the unique G119S mutation in the *ace-1* gene is responsible for OP and CX resistance [5] and the L1014F mutation in the *para*-type sodium channel gene (*kdr-west* mutation) is responsible for pyrethroid resistance in mosquitoes from West Africa [6]. The selection of these mutations leads to increased vector survival in treated environments and potentially to a greater population size, which could increase pathogen transmission. However resistance alleles are often associated with negative pleiotropic effects that lead to fitness disadvantage and reduce resistant allele frequency in the absence of insecticide selection pressure [7]. Population studies in the main African malaria vector *Anopheles gambiae* have suggested a fitness cost associated with *ace-1^R^* and *kdr* mutations [8], [9]. The fitness cost has been characterized, at least in part, for the *ace-1^R^* mutation (increased pupal mortality and reduced body weight [10]) but, to our knowledge, no study has reported a cost associated with the *kdr* mutation so far. Pleiotropy underlining the fitness cost associated with resistance mechanisms is tricky to predict but resistant alleles may alter the physiology of mosquito vectors in various ways and hence may modify the environment of the pathogens they transmit [11]. Indeed, resistant alleles have been demonstrated to influence mosquito susceptibility to infections [12]–[15]. Recently, Howard et al. [16] demonstrated that insecticide resistant *An. gambiae* are more susceptible to infection with the entomopathogenic fungi *Metharhizium anisopilae* and *Beauveria bassiana* than insecticide susceptible mosquitoes. However, no effects of insecticide resistance on the susceptibility of *Culex pipiens* to the avian malaria parasite have been found [17]. Overall, little is known about the influence of the major insecticide resistance mechanisms in other vector-pathogen associations, particularly in the epidemiologically relevant *An. gambiae-Plasmodium falciparum* combination, responsible for a large proportion of the malaria burden. Thus, there is a gap that needs to be filled particularly for implementing malaria control because insecticide resistance has spread to such an extent that it now represents a critical issue.

The most lethal causative agent of human malaria, *P. falciparum* sets up complex interactions to complete its life cycle in the female *Anopheles* mosquito. *Plasmodium* infection leads to cell damage when parasites cross the midgut epithelium [18], [19]; and the activation of immune responses which the parasite may partially be able to evade [20]. Fitness costs associated with insecticide resistance may alter immune system activation, in repairing infection-induced damage and/or enhancing the trade-off with energetic resources used by the parasites for its own development. Most studies on *Anopheles-Plasmodium* interactions have employed the rodent malaria parasite model, *P. berghei*, which is more amenable than *P. falciparum* [21] but differences in infection response between the two model systems suggest close co-adaptation between *An. gambiae* and *P. falciparum* [20], [22], [23].

The present study aims at comparing vector competence towards *P. falciparum* infection between insecticide susceptible and resistant strains of *An. gambiae s.s.* We used three laboratory *An. gambiae* strains sharing a common genetic background, one susceptible to insecticides and two resistants, one homozygous for the *ace-1^R^* mutation and one for the *kdr* mutation, in order to determine the influence of a restricted locus containing the resistant allele on vector competence. *An. gambiae* females were experimentally infected with field isolates of *P. falciparum* and infection levels between insecticide susceptible and resistant strains were compared at the oocyst and sporozoite stages.

## Materials and Methods

### Ethical Statement

Ethical approval was obtained from the Centre Muraz Institutional Ethics Committee under the ethical clearance number 003-2009/cE-cM. All human volunteers were enrolled after written informed consent from the participant and/or their legal guardians.

### Mosquito Strains

Three reference strains of *Anopheles gambiae sensu stricto* S molecular form were used in this study. One is the insecticide susceptible strain Kisumu, collected in Kenya in 1953 [24]. The two other strains were resistant to two distinct class of insecticide: the OP/CX resistant strain Acerkis, homozygous for the *ace-1* G119S mutation and the PYR/DDT resistant strain Kdrkis, homozygous for the *kdr* mutation. Acerkis was obtained by introgression of the resistant *ace-1* G119S allele into the Kisumu genome. The *ace-1* G119S allele was obtained from a sample of a resistant *An. gambiae* population collected in Bobo-Dioulasso, Burkina Faso in 2002 [25]. Kdrkis strain was obtained by introgression of the *kdr-west* allele, harboring the L1014F mutation in the voltage-gated sodium channel gene into the Kisumu genome. The *kdr-west* allele has been obtained from pyrethroid resistant mosquitoes sampled in Kou Valley, Burkina Faso [6]. Resistant strains were obtained through at least 15 successive backcrosses with the Kisumu strain and selection with propoxur insecticide for the Acerkis strain and permethrin insecticide for Kdrkis, so that the three strains share common genetic background at the exception of the locus carrying the insecticide resistance genes [26]. The *ace-1* gene is located in the telomeric region of the chromosome 2R (region 7C) distant from the inversions 2Rb (between sections 11C and 13A) and the *kdr* allele is located on the chromosome 2L (region 20C) distant from the inversion 2La (between sections 27A and 23A) [27]. Kisumu and wild *An. gambiae* from Burkina Faso used for selection of the insecticide resistant strains shared polymorphism for the chromosomal inversions 2Rb and 2La [28], [29]. These inversions are not expected to affect recombination at the selected loci due to low linkage disequilibrium [30]. Insecticide resistant phenotype of all strains was regularly verified using the WHO standard vertical tube protocol [31] with 0.1% bendiocarb and 4% DDT impregnated papers, and the presence/absence of the mutations were checked using the *ace-1* G119S and *kdr* diagnostic PCR [6], [32]. Mosquitoes were kept under standard insectary conditions (27±1°C, 70±8% RH and 12:12 light and dark photoperiod) in the same secure containment facility. Larvae were reared in the same condition at a fixed density (300 first instar larvae in 700 ml of water per tray) and were fed with Tetramin®Fish in order to reduce variation in larval growth rate and mosquito size at emergence. After emergence, adults were fed *ad libitum* on a 5% glucose solution and maintained in 30x30x30 cm cages. One day prior to blood exposition, mosquitoes were starved by removing the glucose solution.

### P. falciparum Experimental Infection by Direct Membrane Feeding Assay

*Plasmodium falciparum* gametocyte carriers were selected by examining thick blood smears from children aged between five and eleven from two villages in southwestern Burkina Faso (Dandé and Soumousso, located 60 km north and 40 km south-east of Bobo-Dioulasso, Burkina Faso, respectively). Parasite quantification in the blood donor was estimated based on an average of 8000 white blood cells (WBC) per µl. Trophozoites (asexual stage non infectious to the mosquito) density was estimated by counting the number of parasites per 100 WBC. When a gametocyte (sexual stage infectious to the vector) was observed, additional fields were added to reach 1000 WBC in order to get more precise quantification. Malaria positive individuals and/or symptomatic participants were referred to the local health center and were treated according to national recommendations. Children with a gametocyte density of more than twenty per µl of blood were selected and a venous blood sample (8 ml) taken after a second confirmation of the gametocyte density. In order to limit the potential effect of human transmission blocking immunity [33], the blood was first centrifuged at 2000 rpm at 37°C for three minutes and the serum replaced with European naive AB serum containing 0.225 UI heparin/ml to prevent clotting. 500 µl of reconstituted blood was added to membrane feeders maintained at 37°C by water jackets. Three to five day-old female mosquitoes of the three mosquito strains were allowed to feed simultaneously on infected blood for up to 30 minutes through a Parafilm membrane. At least three feeders were used for each mosquito strain in order to limit any potential feeder bias. Unfed female mosquitoes were discarded and fully fed mosquitoes of each strain maintained in a large cage (30x30x30cm) under standard insectary conditions on a 2.5% sucrose solution. This procedure was repeated six times, each feeding assay using a different gametocyte-infected blood for oocyst counting and six distinct feeding assays were performed for sporozoite quantitation.

### Oocyst Counting and Measuring of Wing Length

Female mosquitoes from the three strains were dissected on three consecutive days from the 6^th^ to 8^th^ days post-infection (PI) due to the high number of mosquitoes and the time required to dissect out and count oocysts in the mosquito midgut. Each day, one third of the blood-fed mosquitoes were randomly chosen from each cage and midguts were dissected in 0.4% mercurochrome stain and the oocyst burden of each individual female was determined by counting oocysts under a light microscope. The left wing of each female mosquito was cut and was pictured with a dissecting microscope (Leica EZ4D). Wing length was measured from the notch to the wing tip as described by Van Handel and Day [34]. Two measures were performed independently using the ImageJ software (Wayne Rasband, rsb.info.nih.gov/ij/) and correlation between both indicated good agreements (R^2^ = 0.98), thus, the mean of the two measures was used.

### Sporozoite Quantification by Real-time PCR

Females fully-fed on gametocyte-infected blood were maintained in large cages (one per mosquito strain) under standard insectary conditions on a 5% sucrose solution for 14 days. Then the head and thorax were dissected and DNA extracted from each individual mosquito using DNeasy® Blood & Tissue kit (Qiagen). *P. falciparum*-infected individuals were screened by PCR with Pf1/Pf2 primers [35] and positive individuals selected for sporozoite quantification.

A duplex real-time PCR targeting the *P. falciparum* 18S ribosomal gene and the *An. gambiae* ribosomal S7 gene (S7) was used to quantify sporozoite number in individual mosquitoes. We used the *P. falciparum* primers FAL-F and Plasmo2-R described by Shokoples et al. [36] with modifications described by Diallo et al. [37]. For *P. falciparum* quantification, the 18S rDNA gene was amplified from 3D7 gDNA (MR4) using outer primers of the Nested PCR described by Snounou et *al*. [38], [39] and cloned into the pGEM-T vector (Promega) and verified by sequencing [40]. The mosquito gene S7 was amplified as a positive control to ensure that the DNA from the sample was successfully extracted and to later allow normalization when comparing strains and replicates. Specific primers and a corresponding TaqMan probe (FS7: CCAGGATGGCATCGTACAC; RS7: CGCGAGTTGGAGAAGAAGTT; S7probe: VIC–GCGCTCGGCAATGAACACGA-TAMRA) were designed and validated on a serial dilution standard of purified PCR products from the *Anopheles gambiae s.s.* gDNA (efficiency = 99%) [40]. The purified PCR product (396 bp) copy number quantification was performed by spectrophotometric analysis.

The PCR reactions were performed in a final volume of 20 µl containing 5 µl of each purified DNA preparation (10–20 ng), 10 µl of PerfeCTa qPCR FastMix, UNG (Quanta Biosciences), 200 nM of each primer, and 80 nM of each probe. The run was performed with the Rotor Gene 6000 analytical PCR system at 95°C for 5 seconds followed by 60°C for 1 min. The threshold cycle of detection (Ct) was determined for both targets in each sample. Because the amplification of both targets had the same efficiency, the difference in Ct values (ΔCt = Ct_S7_– Ct*_P.f._*) was used to determine the individual relative number of sporozoite genome over that of mosquito genome (Ri = 2^-ΔCt^) allowing comparison of parasite densities between samples. We also calculated the difference in the mean ΔCt of the resistant strain over that of the susceptible strain (ΔΔCt = ΔCt_Kisumu_ – ΔCt_resistant strain_) to compare the relative sporozoite number of resistant mosquito strains over that of Kisumu (R = 2^-ΔΔCt^).

### Statistical Analysis

Infection data consisted of two response variables: either the status of infection, named *infection*: infected (1) or not (0) for each individual, and the number of parasites present in infected individuals, named *burden* either expressed by the number of oocysts per midgut or the individual ΔCt expressing the relative quantity of sporozoite genome over that of mosquito genome (Ri = 2^-ΔCt^). In the analyses, we used five explanatory variables: *strain* (a three-level categorical variable: “Kisumu”, the susceptible reference strain, “Acerkis”, the OP/CX resistant strain and “Kdrkis”, the PYR/DDT resistant strain), *wing length* (a numerical variable), *donor* (a categorical variable, each gametocyte carrier representing a level), *gametocyte density* of the blood donor (a numerical variable, denoted “*gam. density*”) and *day*, the day of dissection (a categorical variable). Statistical analysis was performed using R software (v2.13.2, www.r-project.org). The maximal models included the variables “*geno”*, “*gam. density”* and “*wing”* and their interactions as fixed effects. The “*donor*” and “*day*” variables were used as random variables to account for the nested data structure, *i.e.* the correlation between individuals from the same experimental infection (or blood donor) and the dissecting day within each blood donor. For each analysis, the best random structure was first selected based on the lowest AIC.

Prevalence of infection, *i.e.* the probability of being infected following an infectious blood meal was analyzed using a generalized linear mixed effects model. Two distinct analyses were performed: one corresponding to the prevalence at the oocyst stage (N = 1001 among 6 feeding assays) and another corresponding to the prevalence at the sporozoite stage (N = 526 among 6 feeding assays). For these analyses the response variable (prevalence) was coded 1 for presence of parasite and 0 for their absence. The model was analyzed using the *lmer* procedure (lme4 package, [41]) with a binomial error structure. For this analysis, the random structure with the “*donor*” variable alone gives the lowest AIC. Significance of variables and selection of the minimal model has been assessed using the package *MixMod* [42], which performs a type III hypothesis for the fixed effects of the linear mixed effects models based on the *lmer* procedure (lme4 package, [41]) and computes the *F* statistic based on Satterthwaite’s approximation.

For the analysis of oocyst burden, only individuals that developed at least 1 oocyst were included (N = 734 across 6 feeding assays). As oocyst count may be overdispersed in this system [43], [44], the model was analyzed using the glmmADMB procedure with negative binomial distribution (glmmADMB package, [45]). The nested random structure including the “*donor*” variable alone was selected as described above. The significance of the variables was established using a likelihood ratio test (LRT), which is approximately distributed as a χ^2^ distribution [46], [47].

Sporozoite burden was analyzed using a generalized linear model due to the low number of experimental infections. The ΔCt was used as the response variable and was square-rooted to obtain a normally distributed response variable (N = 171 across 3 feeding assays). Independence and homogeneity of variance were verified [47].

The mean prevalence and intensity of infection among all feeding assays were computed and compared between mosquito strains taking into account for multiple testing using the Bonferroni procedure.

## Results

### Prevalence of Infection

**Figure 1:**
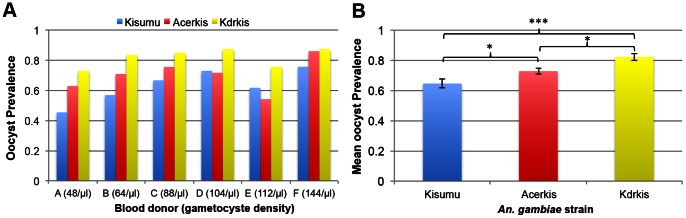
Prevalence of oocysts for each *An.* gambiae strain. Panel A shows histograms presenting the prevalence of oocyst-infected females for each mosquito strain and for each feeding assay. The gametocyte density for each blood donor (/µl of blood) is indicated in brackets. Panel B presents the mean prevalence for each mosquito strain among all 6 feeding assays. Bars above and below the means represent the standard errors of the mean. Tests of significance were corrected for multiple testing using the Bonferroni procedure. Stars indicate the significance level: one star for *p*<0.05; two stars for *p*<0.01; three stars for *p*<0.001.

A total of 1001 females were exposed to 6 infectious blood feeds (encoded A to F) with gametocyte densities ranging from 48 to 144 gametocytes/µl of blood and were dissected 6 to 8 days post-infectious feed to count the number of oocysts in mosquito midguts. Mosquitoes that did not harbor oocysts were classified as uninfected. The prevalence of oocysts per carrier varied from 45.5% to 75.7% in the Kisumu strain; 54.2% to 86.0% in Acerkis strain; and 72.9% to 87.3% in Kdrkis strain (Figure 1A). Table 1 presents the statistical significance of variables influencing the outcome of infection. The mosquito *genotype* significantly influenced the probability of becoming infected upon ingestion of an infectious blood-meal (*F*_df = 2_ = 27.18, *p*<0.001). The *donor* variable has a significant effect (χ^2^_df = 1_ = 9.31, *p* = 0.002) on oocyst prevalence revealing an impact of blood donor distinct from the gametocyte density. Overall, insecticide resistant mosquitoes were significantly more infected than their susceptible counterparts with an average of 82.1% (±2.2) in Kdrkis (Bonferroni adjusted *p*<0.001) and 72.7% (±2.2) in Acerkis (Bonferroni adjusted *p* = 0.048) compared to 64.5% (±2.9) in Kisumu (Figure 1B).

**Table 1:** Statistical analysis of the prevalence of *P. falciparum* oocyst.

Source (fixed)	df	*F*	*p*-value
*genotype*	2	27.18	***p*** **<0.001**
Source (random)	df	?^2^	*p*-value
*donor*	1	9.31	**0.002**

Significance of variables obtained after selection of the minimal mixed effect model is presented, *p-*value<0.05 are bolded.

**Figure 2:**
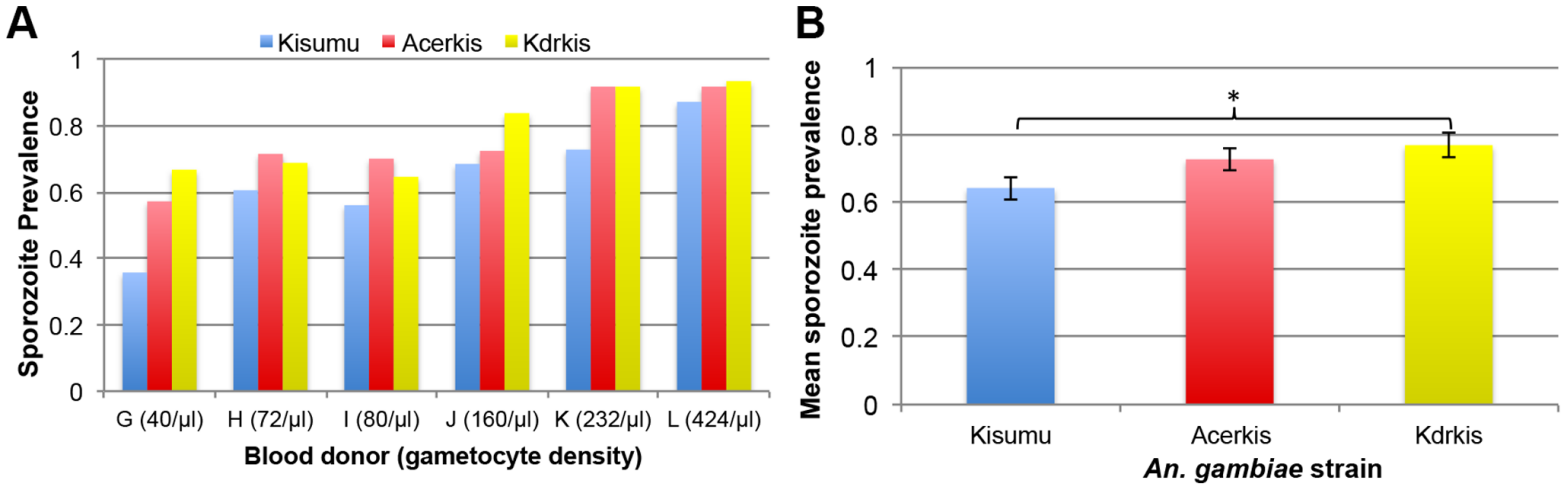
Prevalence of sporozoites for each *An.* gambiae strain. Panel A shows histograms presenting the prevalence of sporozoite-infected females for each mosquito strain and for each feeding assay. The gametocyte density for each blood donor (/µl of blood) is indicated in brackets. Panel B presents the mean prevalence for each mosquito strain among all 6 feeding assays. Bars above and below the means represent the standard errors of the mean. Tests of significance were corrected for multiple testing using the Bonferroni procedure. Stars indicate the significance level: one star for *p*<0.05; two stars for *p*<0.01; three stars for *p*<0.001.

Only sporozoites are able to infect humans from an infected mosquito bite, therefore we measured the prevalence of the sporozoite stage in the head and thorax of the three mosquito strains 14 days after feeding on infectious blood. Data was obtained from six feeding assays with blood containing 40, 72, 80, 160, 232 and 424 gametocytes/µl (named donor G to L, respectively). In the susceptible strain Kisumu, sporozoite prevalence ranged from 35.7% to 87.1% and was lower than that in resistant strains in all feeding assays (Figure 2A): prevalence ranged from 57.1% to 91.6% in Acerkis and from 64.5% to 93.3% in Kdrkis. Statistical analysis revealed a significant effect of *genotype* (*F*_df = 2_ = 5.44, *p* = 0.005) and *gametocyte density* (*F*_df = 2_ = 23.32, *p* = 0.004) on sporozoite prevalence in mosquito salivary glands. However blood *donor* did not impact sporozoite prevalence (χ^2^_df = 1_ = 0.97, *p* = 0.3; Table 2) and so was removed from the minimal model. Overall, the mean prevalence among all infections was 64.1% (±3.4) in the Kisumu strain, 72.6% (±3.2) in the OP/CX resistant Acerkis (Bonferroni adjusted *p* = 0.197) and 76.8% (±3.6) in the PYR/DDT resistant Kdrkis (Bonferroni adjusted *p* = 0.024; Figure 2B).

**Table 2:** Statistical analysis of the prevalence of *P. falciparum* sporozoite.

Source (fixed)	df	*F*	*p*-value
*gametocyte density*	1	23.32	**0.004**
*genotype*	2	5.44	**0.005**
Source (random)	df	?^2^	*p*-value
*donor*	1	0.972	0.3

Significance of variables obtained after selection of the minimal mixed effect model is presented, *p-*value<0.05 are bolded.

### Intensity of Infection

**Figure 3:**
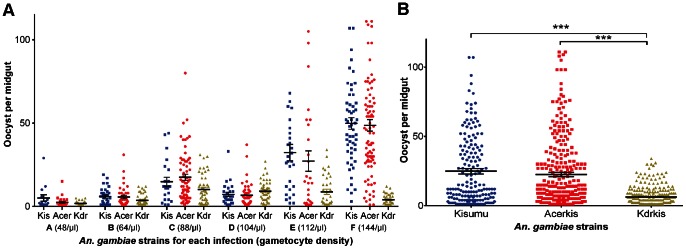
Oocyst burden for each *An.* gambiae strain. Number of oocysts per female midgut is presented as a scatter dot plot for each mosquito strain and for each feeding assay (panel A); and for each mosquito strain among all 6 feeding assays (panel B). The gametocyte density for each blood donor (/µl of blood) is indicated in brackets. Bars above and below the means represent the standard errors of the mean. Tests of significance were corrected for multiple testing using the Bonferroni procedure. Stars indicate the significance level: one star for *p*<0.05; two stars for *p*<0.01; three stars for *p*<0.001.

**Figure 4:**
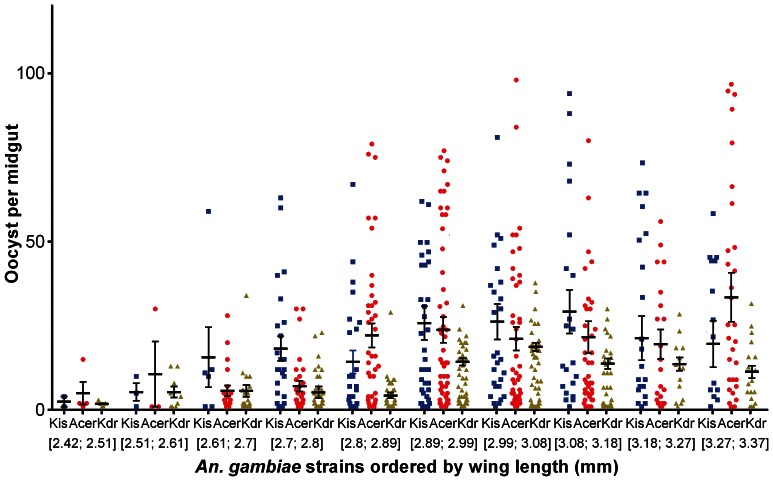
Influence of mosquito wing length on oocyst burden. Oocyst number was presented as a scatter dot plot with the corresponding mean for individuals grouped by wing length. Bars above and below the means represent the standard errors of the mean.

Among the 1001 females dissected for oocyst detection, 734 individuals carried at least one oocyst (N_Kisumu_ = 182, N_Acerkis_ = 296 and N_Kdrkis_ = 256) and were included in the analysis for oocyst burden (Table 3). As for oocyst prevalence, the blood *donor* significantly impacted the oocyst burden (χ^2^_df = 1_ = 37.1, *p*<0.001). The effect of *genotype* was significant (*F*_df = 2_ = 28.24, *p*<0.001) as were the effects of *wing length* (*F*_df = 1_ = 13.14, *p*<0.001) and the effect of *gametocyte density* (*F*_df = 1_ = 6.63, *p*<0.001). A significant *genotype by gametocyte density* interaction was also observed (*F*_df = 2_ = 81.24, *p*<0.001) revealing that the influence of *gametocyte density* was different between mosquito strains. The oocyst burden was significantly reduced in the pyrethroid resistant strain Kdrkis (mean of 6.4±0.4 oocysts/midgut, Bonferroni adjusted *p*<0.001) compared to the susceptible strain Kisumu (24.8±1.9) which was not different to that obtained in Acerkis (22.6±1.6, Bonferroni adjusted *p* = 0,85; Figure 3B). A positive correlation was observed between the oocyst burden and the gametocyte density however this correlation is significant only with the Kisumu and Acerkis strains but not with the Kdrkis strain (Figure 3A). *Wing length* was positively correlated to oocyst burden with larger females harboring more oocysts than smaller females (Figure 4).

**Table 3:** Statistical analysis of the intensity of *P. falciparum* oocyst.

Source (fixed)	df	*F*	*p*-value
*genotype*	2	28.24	***p*** **<0.001**
*gametocyte density*	1	6.63	***p*** **<0.001**
*wing length*	1	13.14	***p*** **<0.001**
*geno.* × *gam. density*	2	81.24	***p*** **<0.001**
Source (random)	df	?^2^	*p*-value
*donor*	1	37.1	***p*** **<0.001**

Significance of variables of the minimal mixed effect model is presented for oocyst burden, *p-*value<0.05 are bolded.

**Figure 5:**
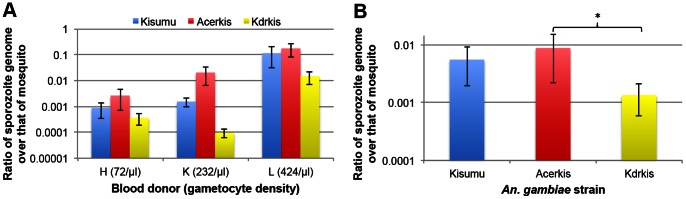
Relative quantity of *P. falciparum* sporozoite genome over that of *An. gambiae* genome. Panel A shows histograms presenting the ratio of sporozoite genome over that of mosquito genome for each mosquito strain and for each feeding assay. The gametocyte density for each blood donor (/µl of blood) is indicated in brackets. Panel B presents the ratio of sporozoite genome over that of mosquito genome for each mosquito strain among all 6 feeding assays. Bars above and below the means represent the standard errors of the mean. Tests of significance were corrected for multiple testing using the Bonferroni procedure. Stars indicate the significance level: one star for *p*<0.05; two stars for *p*<0.01; three stars for *p*<0.001.

Sporozoite burden in the susceptible and resistant strains was assessed by quantifying sporozoite DNA from the head and thorax of individual mosquitoes using a TaqMan real-time PCR assay. Similar amplification efficiency of both targets in each *An. gambiae* strain allowed comparison of the relative sporozoite number between strains. Data was obtained individually from three feeding assays (72, 232 and 424 gametocyte/µl) and thus was analyzed using a generalized linear model (Table 4). The analysis of the sporozoite burden demonstrated a significant effect of mosquito *genotype* (*F_df_*_ = 2_ = 20.31, *p* = 0.0031). We also observed a significant effect of *wing length* (*F_df_*_ = 1_ = 30.07, *p*<0.001). Blood *donor* greatly influenced sporozoite burden (*F_df_*_ = 1_ = 152.4, p<0.001) and this effect appeared significantly different between genotypes (*donor by genotype* interaction *F_df_*_ = 1_ = 19.86, *p* = 0.023). This analysis did not allow for simultaneously testing the effect of gametocyte density. Figure 5A shows the ratio of the number of sporozoite genome over that of mosquito genome and reveals no difference between Acerkis and Kisumu except in the infection with the donor K where the resistant strain had about 10 times more sporozoites than the susceptible one. Kdrkis had a significantly lower sporozoite burden than Kisumu in two infections (donors K and L) but the sporozoite burden was not different between the three strains in the infection with donor H probably due the lower gametocyte density. When all replicates (blood donors) are pooled, sporozoite burden was significantly lower in Kdrkis strain compared to Acerkis strain (Bonferroni adjusted *p* = 0.023; Figure 5B) but not significantly different compared to Kisumu (Bonferroni adjusted *p* = 0.1). In the Acerkis strain, the mean relative sporozoite number appears greater but not significant than that observed in Kisumu due to its higher variability (Bonferroni adjusted *p* = 0.98).

**Table 4:** Statistical analysis of the intensity of *P. falciparum* sporozoite.

Source	df	*F*	*p*-value
*genotype*	2	20.31	**0.003**
*wing length*	1	30.07	***p*** **<0.001**
*donor*	2	152.4	***p*** **<0.001**
*genotype* × *donor*	2	19.86	**0.023**

Significance of variables of the minimal model is presented for sporozoite burden, *p-*value<0.05 are bolded.

## Discussion

We investigated the impact of insecticide resistant mutations in mosquitoes on the development of field isolates of the human malaria parasite, *P. falciparum*, in its main vector *An. gambiae* through experimental infections. As stressed by Lambrechts et al. [48], genetic diversity in natural populations of both vector and parasite can make the study of vector competence complex due to interactions between genotypes. Here, we took advantage of using susceptible and resistant *An. gambiae* strains sharing a common genetic background and also a common polymorphism of chromosomal inversion but differing by the presence of the resistant alleles to specifically discriminate their impact. Thus, any changes in the physiology and life history traits between the insecticide susceptible and the resistant strains must be directly associated with the insecticide resistance alleles.

The influence of blood donor on infection has been tested as a random effect variable because donors were drawn from a large population and they differ in various and unknown ways. The outcome of infections resulting from the different feeding assays varied, as a result of different gametocyte densities between blood donors, and probably other factors like parasite genotypes, gametocyte maturity, gametocyte sex ratio or other blood factors. Overall, the insecticide resistant strains appear to be more susceptible to infection than the insecticide susceptible strain, particularly for the PYR/DDT resistant Kdrkis, for which the parasite prevalence, at both the oocyst and sporozoite stages, was significantly higher. We noticed however that the difference in parasite prevalence between Kdrkis and Kisumu was less pronounced at the sporozoite stage than at the oocyst stage. Prevalence of sporozoite-infected individuals was not significantly different between the susceptible and OP/CX resistant Acerkis strain although oocysts were significantly more prevalent in Acerkis. Because invasion efficiency of sporozoite is very high in natural parasite-vector combination [44], we hypothesize that the difference in infection prevalence at the sporozoite stage (76.8%) compared to the oocyst stage (82.1%) in Kdrkis strain may be due to lower survival of infected individuals harboring the *kdr* resistant allele, although the effect of *P. falciparum* infection on its vector survival remains unclear [49]. The variation of *P. falciparum* prevalence between *An. gambiae* strains lies in the probability of successful development of at least one oocyst. The difference of parasite prevalence in insecticide susceptible and resistant strains may have two non-exclusive origins: the efficacy of transition between gametocyte-to-ookinete in the mosquito midgut in the first hours after blood feeding, which represents the greatest loss of parasite abundance [44] and/or the efficacy of transition between ookinetes to mature oocyst. From the data presented, we are not able to distinguish between an enhanced fertilization or ookinete differentiation neither a facilitated crossing through midgut epithelium in insecticide resistant mosquitoes.

In contrast with the higher prevalence, the parasite burden, (i.e. infection intensity) was significantly lower at oocyst stage in individuals carrying the *kdr* mutation compared to the susceptible strain. At the sporozoite stage this difference was not significant while a trend could be noticed. A lower sporozoite burden could be related at least in part to a lower oocyst burden due to more frequent failure of parasites, without complete clearance, at early development stages in the insecticide resistant mosquitoes (fertilization to young oocysts). In the Acerkis strain, the parasite burden estimated at both oocyst and sporozoite stages were similar to those observed in the insecticide susceptible mosquito colony. Infection intensity was positively correlated to mosquito wing length, a proxy of body size. Statistical analyses of infection took into account the individual wing length to control for its influence on other variable but this effect was not significantly different between strains (Table 2). Then the effect of insecticide resistant alleles is independent from the effect of wing length, which may be due probably to the size of the blood meal as only fully fed females were used.

The mechanism underlying these phenotypes may be numerous as insecticide resistance mechanisms may have direct or indirect influences on the activation of the mosquito immune system and/or its physiology leading to variation in parasite development and survival [11]. Gene expression analysis of pyrethroid resistant *An. gambiae* S-form (metabolic resistance but not *kdr* mediated resistance) revealed up-regulation of *Defensin* and *Cecropin* genes [50], two anti-microbial peptides involved in the anti-*Plasmodium* immune response [51], [52]. If such immune related genes are differentially expressed with the presence of *kdr* mutation or other resistance associated mutations, this may lead to variation of vector competence. A possible explanation of the observed phenotype here may be an alteration of the neurophysiological functions of either the mutated AChE1 or the mutated voltage gated sodium channel. This may disturb the normal process of the insect immune system by interfering with the release of neuro-hormones and neuropeptides that could influence the expression of immune related genes, as previously described in the moth, *Hyalophora cecropia* [53].

Implications of the effect of insecticide resistance on prevalence or intensity of infection on malaria transmission drastically differ. It is worth noticing a contrasting effect of the *kdr* alleles on prevalence and intensity of infection: while parasite prevalence was increased in insecticide resistant mosquitoes, the parasite burden was significantly lower. Prevalence and intensity of *P. falciparum* infection in its natural vector *An. gambiae* were previously shown to be somewhat differentially regulated [54]. Care must be taken to distinguish two different cases: (1) at high infection level (high prevalence and intensity, often due to mosquito exposure to a high gametocyte density), variation in parasite burden is associated with little or no variation in prevalence of infection. This is due to the sigmoidal shape of the relationship between prevalence and parasite burden where prevalence reaches a plateau close to 100% when parasite burden is high [55], and (2) in other cases, where variation in prevalence is not associated with variation in infection intensity [54], [56] indicating that different mechanisms regulate the two modalities. Here, prevalence of infection is higher and parasite burden lower in the mosquitoes carrying the *kdr* allele, which is consistent with the second case where prevalence and intensity of infection are differentially affected.

Variation of *P. falciparum* prevalence (particularly at the sporozoite stage) in *An. gambiae* would have a direct impact on malaria transmission by increasing the proportion of infected mosquitoes in contact with humans. Variation in parasite burden would have little or no impact on parasite transmission because only one oocyst could lead to hundreds of transmissible sporozoites in salivary glands. However the intensity of infection impacts the expression of the vector immune system [57] and may proportionally reduce the fitness of mosquitoes in terms of survival and fecundity [58]. Reduction of vector survival will have a strong impact on malaria transmission [59] because *Plasmodium* parasite has a relative long extrinsic incubation period compared to the mosquito lifespan.

The cost of insecticide resistance in the main malaria vectors has been poorly studied particularly in relation to adult life history traits. Djogbénou et al. [10] has observed a fitness cost associated with *ace-1^R^* on the age and mortality at pupation and on adult dry weight but no cost was found on longevity in an insecticide-free environment. In contrast, little is known about the fitness cost associated with the *kdr* mutation except that *kdr* homozygous individuals appear more susceptible to entomopathogenic fungi [16]. A target site resistance mutation would be expected to impact essential mosquito functions as insecticide targets are components of the nervous system and thus may potentially affect vector behavior. Interestingly, differential expression of salivary proteins involved in blood feeding success (e.g. D7 family) in *Cx. quinquefasciatus* [60] has been associated to the *ace.1^R^* allele, indicating that this resistant allele may interfere with the transmission of pathogens to humans through modification of the vectorial capacity.

Prevalence of insecticide resistance alleles reaches high frequency in West Africa particularly for *kdr*, which is almost fixed in several field populations [61], [62] revealing a strong selective pressure of pyrethroid insecticides. The heavy use of such compounds may have two opposing consequences. On one hand, pyrethroids have been associated with immunotoxic effects in vertebrates, as demonstrated in mice where deltamethrin treatment increased susceptibility to *Candida albicans* [63] and to *Plasmodium berghei* infection [64]. On the other hand, it may impair parasite development especially in resistant mosquitoes, as they are able to absorb a greater amount of insecticides due to an extended contact time to treated materials [65]. Insecticide dose that would not kill resistant mosquitoes may be toxic to the parasite or at least to some parasite strains and further investigations are needed to test this hypothesis. The use of insecticide may be associated with variation in parasite fitness in its mosquito vector thus influencing malaria epidemiology through direct impact on parasite populations. Both insecticide molecules and insecticide resistant alleles would affect malaria epidemiology in a complex way. Moreover, metabolic resistance was not considered in our study but deserves attention as this mechanism is regularly found in combination with target site resistance. Increased detoxification associated with insecticide resistance impacts vector competence and immunity in other vectorial systems and may influence parasite transmission in various ways [11]. Characterization of the vectorial capacity and competence of these newly adapted (resistant) vector populations will shed light on the evolutionary fitness cost on adult life history traits. In addition, further investigations on the evolution of *P. falciparum* virulence in resistant mosquito populations would give valuable insights to predict parasite transmission in response to vector adaptation.

### Conclusions

This study demonstrated that the *kdr* mutation associated with pyrethroid resistance in the major malaria vector *An. gambiae* impacts its competence to transmit *P. falciparum*. The probability of being infected appears higher and the intensity of infection is lower in mosquitoes resistant to pyrethroids than their susceptible counterparts. These results are of great concern for the epidemiology of malaria considering the widespread nature of pyrethroid resistance in Sub-Saharan Africa [66]. Further work is ongoing to identify the determinants of the vectorial capacity and malaria transmission that are affected by these insecticide resistance alleles. Studying the interaction between insecticide resistance and malaria infection in mosquitoes and its evolution will allow us to better understand how parasites have adapted to their local vectors and vice-versa. Answering such question would increase knowledge relevant to develop new control strategies based on mosquito resistance/refractoriness to malaria infection.
